# Earthworms Dilong: Ancient, Inexpensive, Noncontroversial Models May Help Clarify Approaches to Integrated Medicine Emphasizing Neuroimmune Systems

**DOI:** 10.1155/2012/164152

**Published:** 2012-07-25

**Authors:** Edwin L. Cooper, Mariappan Balamurugan, Chih-Yang Huang, Clara R. Tsao, Jesus Heredia, Mila Tommaseo-Ponzetta, Maurizio G. Paoletti

**Affiliations:** ^1^Graduate Institute of Basic Medical Science, China Medical University, Taichung 40402, Taiwan; ^2^Laboratory of Comparative Neuroimmunology, Department of Neurobiology, David Geffen School of Medicine at UCLA, University of California, Los Angeles, Los Angeles, CA 90095-1763, USA; ^3^Division of Vermibiotechnology, Department of Zoology, Annamalai University, Annamalai Nagar-608002, India; ^4^Graduate Institute of Basic Medical Science and Graduate Institute of Chinese Medical Science, College of Chinese Medicine, China Medical University, Taichung 40402, Taiwan; ^5^Department of Biology and Department of Sociology, University of California, Los Angeles, Los Angeles, CA 90024, USA; ^6^Department of Linguistics, University of California, Los Angeles, Los Angeles, CA 90024, USA; ^7^Department of Biology, University of Bari, Via Orabona 4, 70125 Bari, Italy; ^8^Department of Biology, University of Padua, Via U. Bassi, 58/b, 35121-Padua, Italy

## Abstract

Earthworms have provided ancient cultures with food and sources of medicinal cures. Ayurveda, traditional Chinese medicine (TCM), and practices in Japan, Vietnam, and Korea have focused first on earthworms as sources of food. Gradually fostering an approach to potential beneficial healing properties, there are renewed efforts through bioprospecting and evidence-based research to understand by means of rigorous investigations the mechanisms of action whether earthworms are used as food and/or as sources of potential medicinal products. Focusing on earthworms grew by serendipity from an extensive analysis of the earthworm's innate immune system. Their immune systems are replete with leukocytes and humoral products that exert credible health benefits. Their emerging functions with respect to evolution of innate immunity have long been superseded by their well-known ecological role in soil conservation. Earthworms as inexpensive, noncontroversial animal models (without ethical concerns) are not vectors of disease do not harbor parasites that threaten humans nor are they annoying pests. By recognizing their numerous ecological, environmental, and biomedical roles, substantiated by inexpensive and more comprehensive investigations, we will become more aware of their undiscovered beneficial properties.

## 1. Introduction

Animal models especially invertebrates (e.g., fruit flies, nematodes, and earthworms are inexpensive, require less ethical concerns, and are therefore noncontroversial) are crucial to understanding mechanisms that underlie biological processes. These mechanisms are becoming finely tuned where many levels of organization converge from molecular to cellular to organismic and are critically examined revealing a depth never before known. Every animal group and system is being scrutinized revealing levels of biological organization from developmental/functional molecules to organs to systems to organisms. Earthworms have somewhat been crucial in understanding these developmental organizations and are worthy of critical attention for analyzing the nervous, immune and endocrine systems. This paper deals not with questions related to the earthworms' nervous [1], immune [2, 3], and endocrine systems [4, 5]. Instead, the whole earthworm or some of its products has been analyzed in credible experimental research related to biological function in mammals that still require further refinement.

Earthworms are the largest members in the Oligochaeta phylum Annelida or segmented worms, terrestrial relatives of certain marine species, and medicinal leeches that are also of clinical relevance. Earthworms also play essential biological, chemical, and physical roles in ecology. According to Darwin [6], “it may be doubted if there are any other animals which have played such an important role in the world as these lowly organized characters.” Earthworms convert organic matter into rich humus, and improve soil fertility. However, earthworms surprised researchers through their diverse functions beyond improving soil fertility. For example, their behavior and more recently their impressive innate immune potential have captured a new research audience. Darwin comprehensively studied earthworms and became fascinated by the ability of one species *Lumbricus terrestris *   to pull soil litter directly into their vertical burrows. Darwin's earthworm publication *The Formation of Vegetable Mold Through the Action *surpassed his *Origin of Species* in readership during his lifetime. In fact, this book was apparently more popular at the time of its publication than the now better known book on evolution. Only recently have there been attempts to mimic some of these largely environmental observations despite behavioral overtones. [7, 8].

This paper will be divided into three major sections. First, we will review the practices of different cultures that have used or continue to consume earthworms for food as rich sources of protein minerals and fatty acids; whether by design or done intentionally, eating earthworms is still socially unacceptable in most cultures. Second, eating earthworm preparations are often associated with bringing relief to certain ailments. Largely folkloric, these practices offer a formidable array of ideas or leads that will help us to formulate questions that require repetitive validation and necessitate rigorous laboratory investigations. Third, these two approaches although broad with numerous questions to be answered present two examples of emerging results that utilized evidence-based approaches to further expose possibilities for extended experimental analysis.

These directions are outgrowths of and rooted in earlier attempts to decipher inflammation in the whole earthworm (organismic) inspired after Ayurvedic practices in India. The second (cellular, molecular) extends strategies using earthworms from TCM (traditional Chinese medicine) to the nervous system. Attempts to clarify mechanisms where putative remedies exert effects will be reviewed. For example, according to Fan, 1996, lumbrokinase (LK) is a group of proteolytic enzymes, including plasminogen activator and plasmin, separated biochemically from certain earthworm species [9]. Both organismic and molecular analyses are deeply rooted in understanding aspects of inflammation which in turn were originally discovered in relation to bioprospecting and by defining the innate immune system of earthworms [2, 3, 10–12].

## 2. Moving from Food to Cures?

Few people are aware of the earthworm's long association with medicine, despite accounts as early as 1340 A.D. [13, 14]. For example, doctors who practice folk medicine in Burma and India use earthworms for treating certain diseases. The primary use of earthworms in Burma involves treating a disease *ye se kun byo*, characterized by symptoms of pyorrhea (or simply defined as fever). For preparing the cure, earthworms are first heated in a closed pot until they are reduced to ashes. The ashes are then used alone either as a tooth powder or to enhance palatability when combined with roasted tamarind seeds and betel nuts [15], for treating another disease *meephwanoyeekhun thwaykhan*  which affects women with postpartum weakness; this often leaves them unable to nurse their infants. For treatment, earthworms are first boiled in water with salt and onions. The resulting clear fluid is decanted and mixed with the patient's food. Since there is some perceived stigma associated with this treatment, patients are not informed of the medicine's content. Such an example reveals linkages between healing properties of earthworms that are associated with a nutritional component, often the nature of many natural remedies, firmly rooted in one of the oldest disciplines: traditional Chinese medicine (TCM) and similar practices in India referred to as Ayurveda. Now, there emerges more recently recognized practices in the Mediterranean and Middle East.

Herbal medicine is classified into four herbalistic systems: Traditional Chinese, Ayurvedic, Western—which originally came from Greece and Rome to Europe and then spread to North and South America and Traditional Arabic and Islamic Medicine (TAIM). Arabic traditional herbal medicine is still practiced in the Middle East and is acquiring worldwide respect and interest among traditional herbalists and the scientific community. TAIM therapies are successful in healing acute chronic diseases. TAIM attempts to heal infertility, epilepsy, psychosomatic troubles, and depression. In addition, efficacy and safety of TAIM are increasingly important where supervision of techniques and procedures is required for commercial and traditional applications. Still more research is required to solidify the safety and understanding of TAIM and similar therapies [16].

## 3. Attitudes towards Earthworms as Food for Humans

Except for astute farmers and environmentalists, public opinion concerning earthworms is apprehensive or indifferent. For most individuals, earthworms are best understood not a long time ago while dangling from fish hooks! Rozin et al.'s [17] study introduced a scale that measures disgust. The survey's content was focused on giving the word “disgust” a broader semantic representation. Darwin [18] emphasized the word disgust as not only related to bad flavor but also to the cautionary avoidance of ingesting potentially dangerous substances or parasites that have been developed throughout human history. In this context, earthworms would then be related to soil, “waste products of human and animal body.” Psychologists clarified disgust reactions that mainly trigger ingestion reactions in the absence of oral stimuli [19]. Women show a stronger propensity to express disgust but as a natural avoidance of macroparasites. As a result, this becomes a visual aversion to long and slimy animals, to which we associate the innocuous earthworms. To further explore the disgust theory, Prokop and Fančovičová [20] supports the hypothesis that human emotions and behaviors (and aversions) were shaped by natural selection.

These negative conceptions attributed to earthworm consumption are reinforced or pursued in western countries since there are alternative and abundant rich sources of protein; the need to procure earthworms as nourishment would therefore not yield a favorable cost to reward ratio. This aversion is contrasted to tropical and subtropical climates where earthworms, like locusts, are abundant and easily captured by inhabitants as sources of food. Culture plays a vital role in formulation and perpetuation of daily habits and routines. After all we eat through culture or habit more acceptable terrestrial invertebrates such as snails and fresh water crustaceans (crayfish). Without question similar, marine invertebrates (e.g., oysters, clams, shrimp, lobsters) are firmly rooted as enormously successful delicacies.

### 3.1. Southeast Asia, Middle East and Africa

In addition to food sources, since medicinal properties of earthworms are relatively widely used, it is noteworthy to describe practices in many other countries and cultures. In Burma and Laos, for instance, earthworms have been used to treat smallpox (replaced in the 20th century by vaccines derived from evidence based approaches). To begin a therapeutic preparation, earthworms are first soaked in water and patients are then bathed in the resulting liquid. Next, the worms are then roasted, powdered, mixed with coconut water, and consumed by the patient. Such a treatment hastens the disease's severity thereby may be the ultimate cause for reducing mortality by 75 percent [21].

Earthworms are appreciated in most eastern and southeast Asian countries. In ancient China, they have been eaten in Fujian and Guangdong provinces [24] and appeared among the special foods on Island of Hainan, where they were cooked in pieces of bamboo (*P'ing-chou k'o-t'an*) [25]. Even now, in Taiwan, Hainan, and Guangdong, earthworms are considered a delicacy [24, 26, 27]. In conjunction with Laos and Siam, other arthropods and earthworms are part of traditional Chinese medicine [27, 28]. Ljungström and Reinecke [29] report of van Hass, a German marine biologist, who had been offered an earthworm pie in Japan. A Dutch traveler, at the border between Transvaal and Botswana, met elderly African man eating earthworms that had been knotted on a stick and roasted over an open fire.

Earthworms are considered a potent and effective remedy in Iran. In this culture, earthworms are baked and eaten with bread to reduce bladder stones which are expelled after the meal. As still another example, earthworms are also dried and eaten to cure yellow skin in patients with jaundice. Concerning alopecia, or hair loss, earthworm ashes are reported to assist regrowth by applying them on to the scalp with rose oil [15].

### 3.2. North America

According to Native Americans, Carr described how Cherokee Indians used earthworm poultices to draw out thorns; here is the account: “Just make your poultices of chopped up worms that are powerful” [30]. Among the Nanticoke Indians of Delaware, earthworms have been known to serve as a remedy that alleviates pain due to rheumatism [30, 31]. According to one graphic description, affected members would “put fishing worms in a bottle and then apply to stiff joints despite the resulting foul order, there is a measure of alleviating pain” [30]. Concerning mechanisms, information is scanty, but biochemists suggest that earthworm lipids that contain fatty acids play a crucial role during therapy. And on another subject other researchers have also isolated a bronchial dilating substance from earthworms; this is related to reports of earthworm extract that destroys blood clots—the commercial material known as lumbrokinase Cooper and Yamaguchi [32]. Despite long standing information from the lore of Native Americans, much is only becoming minimally apparent just recently in the mainstream medical world, more as a quirky and misunderstood brand of folklore. We must recall however that modern western medicine experienced similar humble origins, long before the advent of evidence-based medicine.

### 3.3. Australia and New Zealand

Benham [33] refers to the Maoris' consumption of earthworm and connects them to earthworm's distinctive names for earth that contain worm (*toke.*). Best [34] enumerated eight different kinds, (*Kuharu, Noru, Wharu, Tarao, Pokotea, Tai, Kurekure, Whiti*). The latter two are known for their sweet and residual flavor lasting for a couple of days. These were offered to chiefs and given to a dying person as the last food (*o matengo*). Maori formerly put earthworms in a bowl, to be cooked in water warmed by means of hot stones, and then preserved in gourds. Australian aborigines incorporated earthworms and insects as additional dietary sources [35]. Some of them have also been considered among their totemic animals. The Aranda aborigines are known to devote to earthworms a special *corroboree*  to promote rebirth of this species, vital for their subsistence. During this ceremony, small round stones that represent *earthworm *cocoons are thrown from a rock to propitiate earthworm multiplication [36]. In Papua New Guinea, earthworms have been eaten by nomadic people along the Salumei River, an affluent of the Sepik [37]. Meyer-Rochow [38] reports that some New Guinea groups, known for their homosexuality, like the Onabasulu, the Kaluli, and others, showed aversion for any organisms such as earthworms living in soil. Earthworms can also be consumed as a form of protein, containing amino acids, minerals, fatty acids, and trace elements [39]. Among the Ye'Kuana and the Piaroa native people of Alto Orinoco or among the Maori of New Zealand, earthworms are widely consumed for their nutritional content and more relevant studies suggest that earthworm consumption may be useful as protein supplements.

### 3.4. South America

In South America, many different invertebrates are important food, especially in the Amazon. The Ye'Kuana tribe from Alto rio Padamo, Venezuela (Amazonas), considers earthworms as a delicacy. The municipality of Alto Orinoco's earthworm's market rates of consumption are three times that of fish and other animal meats. The Amazonian tribe eats predominately two species of earthworms and recognize sixteen “ethnospecies” (Table 1) [23] using different names. Earthworms as gourmet (*motto *and *kuru*) food for the Ye'Kuana are prescribed for women during the first month following childbirth. Pregnant women from this group consume a diet predominately composed of cassava and earthworms [39]. Convalescent and anemic people also consume these sweet annelids [22, 23]. Ye' Kuana's interest in earthworms is evident by their praising motto (*Andiorrhinus* *motto*) distribution. Earthworms for consumption are collected as adults and cocoons during April-May from riverbanks and introduced into stream banks, in which they are absent, to increase the success of dissemination, revealed by observations made in Ye'Kuana villages in the lower rio Padamo. The Piaroa Indians living in Alto Orinoco only eat earthworm species they call *wua' * (*Andiorrhinus* *motto.)*

### 3.5. Earthworm's Natural Content

Earthworms have been viewed as an important protein source. Sun et al. [41] confirmed high protein content consisting of 78-79 grams of free amino acids per liter. There is a high content of vitamins and minerals, particularly iron (Fe) and calcium (Ca). Paoletti et al. [39] analyzed earthworm's potential as a source of protein, nutrients and fatty acids for human consumption (Table 2) [23]. By examining the diet of Amerindians of the Amazonas (Alto Orinoco) in Venezuela, results revealed the consumption of leaf and litter-feeding invertebrates as a means of recovering protein, fats, and vitamins. Thus, a new perspective was proposed for developing sustainable animal food production while retaining biodiversity. In effect, the *kuru *and *motto *earthworm species consumed by the Ye'Kuana (or Makiritare) contain useful quantities of calcium, iron fatty acids and other nutrients essential to the health of those who consume them (Table 3) [23].

## 4. Ayurveda: Approaches to Biomedicine Using Earthworms

### 4.1. Introductory Comment

One Ayurvedic theory asserts that each human possesses a unique combination of dosas that define that person's temperament and characteristics [42–44]. Another view, also present in the ancient literature, asserts that humoral equality is identical to health, and those persons with preponderances of humours are proportionately unhealthy, and not their natural temperament. In this current paper, emphasis will be placed on the whole animal, the laboratory rat. Various functions are modified by using extracts and/or paste derived from earthworms to ameliorate certain experimental inflammatory responses. The subject of Ayurveda is too extensive; a cursory statement here emphasizes the beginning revealing similarities to TCM.

### 4.2. Earthworm Paste Alters Inflammatory, Oxidative, Hematological, and Serum Indices

Ayurveda is a system of traditional medicine native to India practiced since the mid- second millennium BCE. Ayurvedic practitioners have developed for many years several medicinal preparations and surgical procedures for relieving ailments. Ayurvedic use of earthworms has revealed biological mechanisms and guided initial approaches for understanding integrative medicine. Certain biomedical properties of earthworm paste have been analyzed by Cooper et al. [46] revealing alterations in inflammatory, oxidative, hematological, and serum biochemical indices derived from inflamed rats. Throughout these descriptions, distinctions between paste and extract are not clear. Experiments have focused on the Wister albino rat (*Rattus norvegicus*) therapeutic anti-inflammatory, antioxidative, hematological, and serum biochemical markers associated with earthworm paste (EP) from *Lampito mauritii *  (Kinberg), when compared with the standard doses of the well-known anti-inflammatory drug, aspirin. Earthworm paste was administered at 80 mg/kg to rats in which inflammation has been induced. According to prediction, there was a reduction of inflammation, restoration of levels of antioxidants-reduced glutathione, glutathione peroxidase, superoxide dismutase, catalase, and thiobarbituric acid reactive substances. Concerning cells, there was normalization of erythrocytes and leukocytes numbers, as well as differential levels of neutrophils, lymphocytes, eosinophils, hemoglobin, and serum biochemical content and acid electrolytes. Finally, increased amounts of polyphenolic content within earthworm tissues, were attributed to administration of earthworm paste.

### 4.3. Antiulceral and Antioxidant Properties of Earthworm Paste

About the same time as the previous work, Prakash et al. [48] analyzed antiulceral and antioxidant properties of earthworm paste in the same earthworm species, *Lampito mauritii *(Kinberg). Similar to the earlier work, investigators analyzed in conjunction with the standard antiulceral drug ranitidine on Wistar strain albino rats *Rattus* *norvegicus*. Ranitidine is used to treat ulcers; gastroesophageal reflux disease (GERD), a condition in which backward flow of acid from the stomach causes heartburn and injury of the food pipe (esophagus); conditions where the stomach produces too much acid, such as Zollinger-Ellison syndrome. After rats were administered 200 mg/kg aspirin, there were increased volumes of gastric juice secretion, total and free acidity and ulcer index, but reduced pH levels. In contrast, there were reduced antioxidant levels, but increased levels of thiobarbituric acid reactive substances. Furthermore, ulcer induced rats showed enhanced pH, decreased volume of gastric juice, free acidity, total acidity, and a reduction in ulcer index when ranitidine was used simultaneously with earthworm paste. Over-the-counter ranitidine is used to prevent and treat symptoms of heartburn associated with acid indigestion and sour stomach. Ranitidine belongs to a class of medications called H_2_  blockers. It decreases the amount of acid synthesized in the stomach. Activities of reduced glutathione, glutathione peroxidase, catalase, superoxide dismutase increased while thiobarbituric acid decreased. Results were significantly more pronounced in rats that had been administered a higher dose of earthworm paste/kg, suggesting that antioxidative properties at higher dosages of earthworm paste exert a more significant therapeutic effect than ranitidine.

### 4.4. Preliminary Clues: Does Earthworm Extract Exert Hepatoprotective and Antioxidant Properties?

Clearly, there is a need for a plausible explanation worthy of further investigation that targets well-defined mechanisms. At another level, Balamurugan et al. [49] examined hepatoprotective and antioxidant properties when they tested earthworm extract (not clear of difference if any between paste and extract?) against paracetamol-induced liver injury and compared against silymarin. Silibinin (INN), also known as silybin, is the major active constituent of silymarin, standardized extract of milk thistle seeds, containing a mixture of flavonolignans that consist of silibinin, isosilibinin, silicristin, and silidianin. Silibinin itself is a mixture of two diastereomers: silibinin A and silbinin B in approximately equimolar ratio. Both *in vitro* and research using animals suggest that silibinin possesses hepatoprotective (antihepatotoxic) properties that protect liver cells against toxins. Silibinin has also been demonstrated to contain anticancer effects against human prostate adenocarcinoma cells, ectocervical carcinoma cells, colon cancer cells as well as small and larger lung carcinoma cells.

Applications of earthworm extract caused a reduction in liver antioxidants, glutathione peroxidase, and catalase in serum components: total protein, alkaline phosphatase (ALP), aspartate aminotransferase, alanine aminotransferase, bilirubin and liver thiobarbituric acid reactive substances. Alterations have been attributed to thiobarbituric liver injury induced in paracetamol-administered rats. Increased activities of liver GSH, SOD, GPx, CAT, and total protein levels but decreased contents of serum ALP, AST, ALT, bilirubin, and liver TBARS were observed in rats administered with different doses of earthworm extract, 100, 200, and 300 mg, that suggest similar effects.

### 4.5. Anti-Inflammatory and Antipyretic Activities of Extract

Moving further, Balamurugan et al. [50] conducted more experiments designed to better understand therapeutic anti-inflammatory and antipyretic therapeutic properties of crude earthworm extract (EE) (there is still a need to define the differences between extracts and pastes). Wistar albino rats, *Rattus norvegicus * underwent induction of an inflammatory response by injecting histamine into their hind paw. A granuloma and pyrexia were also induced by Brewer's yeast (a granuloma is a collection of immune cells, e.g., macrophages that form when the immune system attempts to isolate or wall off but unable to eliminate substances perceived as foreign). Such irritating substances include infectious organisms, for example, bacteria and fungi as well as other materials like keratin and suture fragments. Moreover, granuloma is therefore a special type of inflammation that can occur in a wide variety of diseases. The adjective granulomatous means *characterized by granulomas*. Pyrexia or fever is a common medical sign characterized by an elevated temperature above the normal range of 36.5–37.5°C (98–100°F) due to an increase in the normal regulatory set-point. This increase in set-point triggers increased muscle tone and shivering. As an example, effects of anti-inflammatory drug-indomethacin and antipyretic drug paracetamol have been compared. Administration of indomethacin, paracetamol, and/or different doses of earthworm extract (50, 100, and 200 mg) restored parameters to normal conditions of histamine, turpentine-induced inflammation, and Brewer's yeast-induced pyretic reactions. These encouraging and confirmatory experiments have been considered successful by interpreting a dose-dependent delivery of extracts to Wister albino rats. There were significant (1) inhibitions of paw edema and granuloma; (2) reductions in hyperpyrexia when rats were treated with both standard drugs and different doses of EE. These results offer significant evidence for anti-inflammatory and antipyretic components of whole earthworms. Moreover, they appear similar to those induced by a more purified earthworm glycoprotein complex (G-90) that has been isolated and tested [51].

## 5. Earthworms Influence TCM Approaches to Nervous System Development and Function

### 5.1. Introductory Comment

TCM appears to be more dominant in the world of early medical practices despite the prominent lore of India. Dilong, earthworm (*Pheretima aspergillum*), is associated with cold and influences the bladder, liver, lung, and spleen channels. Earthworms stop spasms, reduce heat toxins, settle wheezing, promote urination, and unblock and activate those channels where earthworms are commonly used to treat high fever with convulsions, swollen and painful joints, long-term cough, difficult urination, and high blood pressure.

### 5.2. Dilong or Earthworm: Its Role in Peripheral Nerve Regeneration after Injury

We turn now to the Chinese equivalent of integrative medicine practices, comparable to traditional chinese medicine (TCM) approaches to nervous system development by using earthworms, referred to as Dilong in Chinese literature which has been analyzed to understand other biomedical outcomes. They are more widely known than usages in Ayurvedic practices. Schwann cells (shwän, shvän) are cells that cover axons in the peripheral nervous system and form the myelin sheath.

Earthworms with intact nervous systems can regenerate amputated body parts. Reviewing this, Wei et al. [52] examined regeneration of nerve cells in the presence of *Lumbricus * (earthworm) extract. In TCM, using *Lumbricus*  is one method employed for millennia in China to promote nerve function. To test this, nerve function was surgically impaired in Sprague-Dawley (SD) rats by clamping the left sciatic nerve. As essential for scientific value, sham-operated groups (surgery but no sciatic nerve clamping), nontreated control groups, and treatment groups were administered 2 mL 0.9% NaCl, 0.9% NaCl, and *Lumbricus *  extract (1 g/mL). Treatments were administered for six weeks once daily after operations. During this period, motor and conductor functions of injured sciatic and regeneration of myelinated nerve were assessed by immune histochemistry. Three important results were evident (1) Nerve function index value, treatment group was higher than in controls. (2) For conduction velocity of injured sciatic nerve, treatment group was higher than in controls at weeks 3 and 6. (3) For the number of regenerated myelinated nerve fibers in the treated groups were higher than controls at weeks 2 and 6.

To begin another approach, at the more molecular level, Chang et al. examined earthworm-induced phosphorylation of ERK1/2 and p38, but not JNK, activated downstream signaling expression of PAs and MMPs in a time-dependent manner [40]. Earthworm effects were also shown to stimulate ERK1/2 and p38 phosphorylation. However, it was attenuated by pretreatment with U0126 and SB203580, resulting in migration and inhibition of uPA-related signal pathway. These results have been confirmed using small interfering ERK1/2 and p38 RNA, suggesting that earthworm product can stimulate Schwann cell migration and upregulate PAs and MMP2/9 expression mediated through the MAPK pathways, ERK1/2 and p38. Together, this information suggests that the MAPKs (ERK1/2, p38), PAs (uPA, tPA), MMP (MMP2, MMP9) signaling pathway of Schwann cells can be regulated by earthworm product that could be important in Schwann cell migration and nerve regeneration.

### 5.3. RSC96 Schwann Cell Proliferation and Survival Induced by Dilong through PI3K/Akt Signaling Mediated by IGF-I

**Figure 1:**
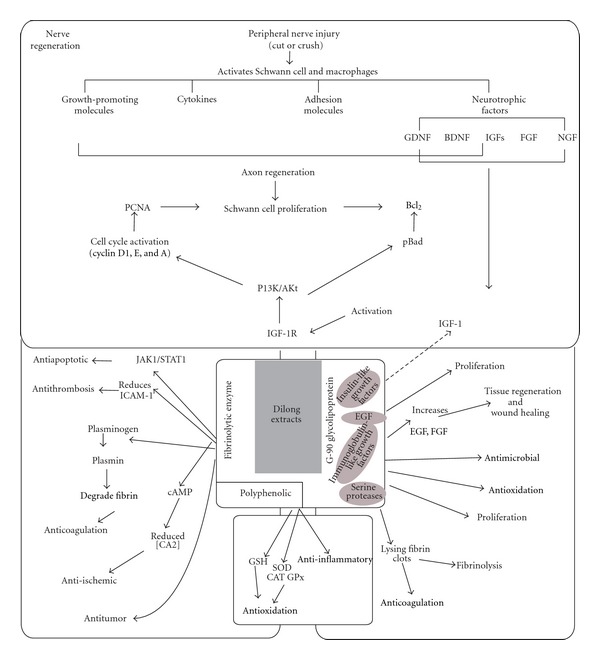
Schematic model of the survival and proliferative effects of Dilong extract on RSC96 Schwann cell. Stimulation of Schwann cell with Dilong extract activates IGF-I signaling, leading to upregulation of the PI3K/Akt pathway and activation of the cell cycle regulatory proteins cyclin D1, E, and A, resulting in the survival and proliferation of RSC96 Schwann cell. Dotted lines indicate the hypothetical molecular mechanism of the bioactive compound present in Dilong powder (from [40]).

Going steps further, another investigation by Huang et al. [53] analyzed Dilong-induced PI3K/Akt signaling pathway as mediated by IGF-I. Experiments focused on molecular mechanisms that promote dilong neuron regeneration. Treatment with extracts of Dilong-induced phosphorylation of the insulin-like growth-factor-I- (IGF-I-) mediated phosphatidylinositol 3-kinase/serine-threonine kinase (PI3K/Akt) pathway. It also activated protein expression of cell nuclear antigen (PCNA) in a time-dependent manner. By means of cell cycle analysis, G(1) transformed into S phase in 12–16 h, and S transited into the G(2) phase at 20 hours after exposure to earthworm extract Figure 1.

### 5.4. Earthworm Extracts Facilitate PC12 Cell Differentiation and Promote Axonal Sprouting in Peripheral Nerve Injury

 Chen et al. [54] have performed *in vitro* and *in vivo* effects of another earthworm *Pheretima aspergilum *on peripheral nerve regeneration. Findings indicated that earthworm water extracts caused a significant enhancement of nerve growth factor-mediated neurite outgrowth from PC12 cells and expressions of growth-associated protein 43 and synapsin I. By *in vivo* analysis, silicone rubber chambers filled with EW extracts were used to bridge a 10 mm sciatic nerve defect in rats. After eight days after implantation, the earthworm water extract groups exhibited a higher success percentage of regeneration (90%) compared to controls (60%) that received saline. Quantitative histology of successfully regenerated nerves revealed that myelinated axons in EW group at 31.25 microg/mL were significantly more than those in controls (*P* < 0.05). These results suggest that EW extracts may serve as a potential growth-promoting factor for regenerating peripheral nerves.

**Figure 2:**
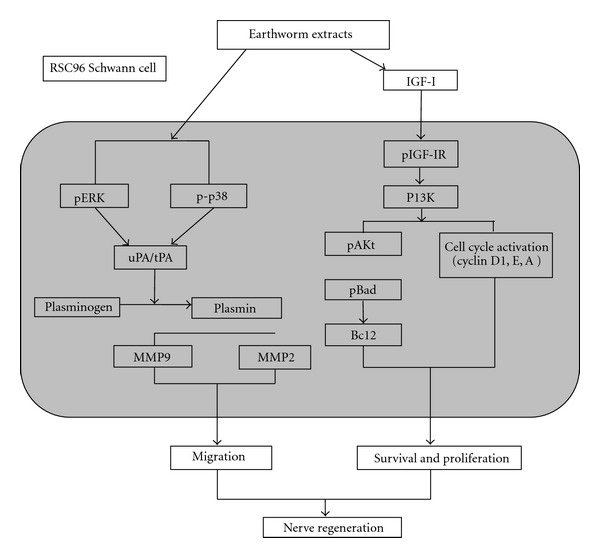
Schematic model of migrative survival and proliferative effects of Dilong extract on Schwann cell (From [45]).

Chang et al. [45] concluded that Schwann cell migration and proliferation play critical supportive roles in regeneration of injured nerves. Dilong is widely used in Chinese herbal medicine to remove stasis and stimulate wound-healing functions. However, the molecular mechanisms induced by Dilong in Schwann cells that causes their migration and proliferation remain unclear. This investigation showed mechanisms that included (i) migration signaling, MAPKs (mitogen-activated protein kinases), mediated PAs and MMP2/9 pathway; (ii) survival and proliferative signaling, IGF-I-(insulin-like growth-factor-I-) mediated PI3K/Akt pathways and (iii) cell cycle regulation were analyzed and identified. Clearly dilong stimulates RSC96 cell proliferation and migration, inducing phosphorylation of ERK1/2 and p38, but not JNK, and activates downstream signaling expression of PAs (plasminogen activators) and MMPs (matrix metalloproteinases) in a time-dependent manner. Dilong also stimulated ERK1/2, but p38 phosphorylation was reduced by pretreatment with chemical inhibitors (U0126 and SB203580) and small interfering ERK1/2 and p38 RNA. These results support and trace the unknown RSC96 cell migration and proliferation mechanism that appear to be induced by Dilong and demonstrate the molecular potential of Dilong as a candidate for clinical applications to nerve regeneration Figure 2.

## 6. Antibacterial Agents and in Prophylactic Molecules without Apparent Relationship to Ayurvedic nor TCM Approaches?

There are many earthworm studies that seem to be not related to either Ayurveda or TCM based on antibacterial agents and prophylactic molecules that now have wider implications. Cooper et al. [11] discovered that earthworms have been prominent with respect to lysis of bacteria and with other implications to disease. The two molecules, lysenin and eiseniapore, are dependent on intracellular lipid trafficking mechanisms. Trafficking dysfunction leads to disease development, such as Tangier disease and Neumann-Pick disease type C, or contributes to the pathogenesis of Alzheimer disease and atherosclerosis. Lysenin reacts with fibroblast membranes from patients with Niemann-Pick disease, an interesting finding, and one that may shed some light on clinical relevance. The binding of lysenin to sphingomyelin on cellular membranes serves as a useful tool in probing the functions of sphingomyelin in biological membranes and in explaining the mechanisms of lysis in earthworms. These results show the relevance of concerted analyses in lytic pathways to demonstrate earthworm-mediated effects on the immune-defense system. Studies featuring earthworms as a source of antibacterial agents increase, should not be surprising. We must remember that earthworms inhabit soil and have evolved powerful survival mechanisms [55]! Their relevance to treating human diseases becomes a worthwhile and promising challenge.

## 7. Recapitulation and Perspectives

This paper depicts earthworms as well-versed organisms, superb animal models that have contributed to the emergence of the field of complementary and alternative medicine, sometimes referred to as integrative medicine. The apparent limited roles that earthworms play in nutrition must be differentiated from equally important medicinal applications. With more examination, the innate immune system of earthworms has allowed immunologists to better understand both cellular and humoral responses to create natural and medical substances that reduce inflammatory responses, considered by many to be at the root of many diseases [11, 47, 56, 57] (Table 4). Thus, there are notable prospects for expanding TCM and Ayurveda [58–60].

This work contributes significantly to understanding that what we eat may affect health. Actually some of the work reported here calls to mind pioneer experiments by Levi-Montalcini and the first discovery of nerve growth factor (NGF) [60]. Although there remains much to be done using products from earthworms, the beneficial effects remain reasonably clear after centuries of use by ancient cultures. Moreover, we should mention if only briefly certain benefits derived from other invertebrates including the long history of honey bees [61, 62], maggots [63], and the leech a near invertebrate of the earthworm [64]. Including the earthworm, they have been commercially exploited for human benefit, none of them are pests, parasites nor vectors of disease, and none present expensive and time-consuming ethical concerns.

## Figures and Tables

**Table 1:** List of earthworms (Glossoscolecidae) ethnonames from two Ye'Kuana villages in the Alto Rio Padamo area, Amazonas, Venezuela as reported by Paoletti and Dufour [22, 23].

Earthworms ethnonames	Characteristics
Motto *(Andiorrhinus motto) *	White, lower river banks, edible
Daicik	White small lower river banks
Vejaj	White medium size
Toccamo, Taegic, Modoiddi,	White
Mouato Araito, Cetoka Mawada, Kurujicette, Canaje	Not available
Kuru *(Andiorrhinus kuru) *	Red-brown 40–60 cm, edible, in forest
Saridi	40–50 cm dark brown, in forest
Sciciu and Catasu	Only for line fishing white-pink 6 cm upper river bank

**Table 2:** Proportion of insects, spiders, and earthworms as percentage of animal protein in Amazonian diets of Amerindian communities as adopted by Paoletti and Dufour [22].

Yanomamo (Alto Orinoco, Venezuela)	1–3% annually as mentioned by Lizot underscore the real amount eaten
Tukanoan Indians (from Vaupes, Columbia)	12% for men's diet, and 24% of women's diet
Piaroa, Rio Cuao (Alto Orinoco, Venezuela)	8% annually, Zhenjun, S. (1992)
Guajibo (Alto Orinoco, Venezuela)	60–70% during the rainy season

**Table 3:** Mineral content (*μ*g/g dry weight) of *motto* and *kuru* as adopted by Paoletti and Dufour [22].

Mineral	*kuru* body (*n* = 1)	*kuru* gut organs^a^ (*n* = 1)	*motto* body (*n* = 1)	*motto* smoked (*n* = 7)	
				mean	S.D.
Aluminium	1430	36200	5220	2640	962
Arsenic	0.91	0.53	1.41	1.41	0.23
Calcium	2650	12900	7070	1020	260
Chromium	30.5	141	90.1	1.67	0.56
Copper	5.63	6.23	8.17	10.9	6.2
Iron	1050	12000	2990	1080	121
Potassium	3430	4510	897	6810	599
Magnesium	527	457	792	730	52
Manganese	17.9	29.8	74.6	22.6	2.7
Molybdenum	0.61	1.6	1.41	0.29	0.05
Sodium	997	1240	548	2160	116
Nickel	10.6	53.2	38.6	0.64	0.14
Phosphorus	3500	4220	3560	5620	326
Lead	4.72	ND	2.5	4.17	1.43
Selenium	9.02	ND	ND	2.71	0.41
Strontium	7.43	27.2	28.8	4.12	1.07
Vanadium	1.25	19.8	5.09	0.8	0.2
Tungsten	1.49	0.92	1.31	1.51	0.33
Yttrium	0	0.52	3.74	3.09	0.81
Zinc	149	93.5	131	96.3	15.4

^a^Parts not eaten; ND: not detectable.

**Table 4:** Functions of lumbrokinase.

(i) Dissolve clots and protect against ischemic heart disease and stroke.	
(ii) Lower fibrinogen levels in cancer patients, which is strongly associated in scientific studies with better outcomes, less metastasis, and slower growth of tumors.	
(iii) Dissolve bacterial biofilms present in chronic infections in conditions like autism and lyme disease allowing antimicrobials to work effectively.	
(iv) Offer antiplatelet, antithrombotic, and antiapoptotic activity, remarkably regulating hypercoagulations.	

Adapted from a table from reference as adopted by Cooper [47].
